# Assessing thermal resistance of wet suits on human subjects during aquatic activity by a heat flux method

**DOI:** 10.1186/2046-7648-4-S1-A66

**Published:** 2015-09-14

**Authors:** Bernard Redortier, Emmanuelle Brossard, Remi Tillol, Remi Goffinet

**Affiliations:** 1 Decathlon SportsLab, Thermal Comfort Laboratory, Villeneuve d'Ascq, France

## Introduction

Thermal protection of wet suits for aquatic sport (surfing, snorkelling, outdoor swimming) is impacted by design (stretch and fit, type of seams, design of openings) as water coming in and flushing between the skin and suit dramatically increases body heat loss. We developed a test protocol for measuring thermal resistance of a suit on a human subject while performing an activity in water. Six surf suits were compared and two swimming suits differing by thickness and stretch of neoprene, type of seams and neck design.

## Methods

The test is operated in a swimming pool 1.3m deep, equipped with a counter-current flow system and an indoor rower fixed on the bottom. Water temperature is between 18 and 24°C, no regulation required.

Ultra-thin and sensitive heat flux sensors (Captec, size 10mmx 10mmx 0.6mm, 3μV.W^-1^.m^-2^) measuring heat loss (W.m^-2^) and temperature (°C) are taped on skin of a human subject (19 sensors in total: torso front 5, torso back 5, arm 4, leg 5). The subject is then dressed with the suit, plus when required shoes + gloves, mask + scuba. After entering the water the subject exercises for 10 minutes: either rowing fully immersed for testing surf / snorkelling suit, or front crawl swimming for swimming suit; the rowing exercise elicits movements of all body segments and favours water entrance in the back, while being easy to standardise. Then the subject stays immersed at rest in still water for 8 minutes. Measurements are integrated over the last three minutes of active and rest condition into regional (arms, legs, torso front, torso back) and whole body thermal resistances. The experiment is replicated with 6 subjects.

## Results and discussion

(1) Whole body resistance (95% CI ≤ 10%) ranked the suits in agreement with usual perception in real use, validating the realism of the test protocol.

(2) The difference on thermal resistance between active and rest condition is due to water entrance into the suit induced by activity, which we characterised by a leak resistance (m^2^.K.W^-1^) in parallel with resistance of rest condition, out of which an effective leak flow (mL.min^-1^) was calculated. For surf suits, legs and arms did not show significant water leak, as the suit tightly fitted the limbs. On the torso for the suit with sewed seams a leak flow of 60mL.min^-1 ^was found (turning into a 20% loss of thermal resistance), 30mL.min^-1 ^for similar style but with glued seams, 15mL.min^-1 ^for improved style and stretch, 4mL.min^-1 ^for top of the range front zip design. For swimming suits, for which openings at neck and cuffs faced the water flow, more than 50% of the thermal insulation was lost by water flushing in the back, 35% for arms. High stretch neoprene, which is required for ergonomics reasons, was found to favour water entrance and to be a negative factor for thermal protection during the swimming phase.

## Conclusion

Besides an assessment of thermal insulation for comparing suits or calculating their temperature range of use, the method also quantifies water flush for each body segment and by how much conceptual and design features impact insulation.
